# ﻿Spatial activity and sheltering behaviour of terrestrial isopods (Isopoda, Oniscidea): a field experiment

**DOI:** 10.3897/zookeys.1225.125030

**Published:** 2025-02-05

**Authors:** Romana Fialová, Ivan Hadrián Tuf

**Affiliations:** 1 Department of Ecology and Environmental Sciences, Faculty of Science, Palacký University Olomouc, Olomouc, Czech Republic Palacký University Olomouc Olomouc Czech Republic

**Keywords:** *Armadillidiumversicolor*, circadian activity, locomotion, marking, *Porcellioscaber*

## Abstract

A field study of spatial activity and sheltering behaviour of terrestrial isopods was carried out using *Porcellioscaber* and *Armadillidiumversicolor*, two model species of woodlice. Individuals of both species were colour-marked and released into an area with artificial shelters, and their behaviour was then observed for several days. Vagility of both species was found to be similar; their dispersal ability was measured to be at least 1.1 m/h. The number of animals recaptured declined over time as they left the field-experiment area. The provided shelters were found to be unevenly inhabited both in relation to their position and the time elapsed since the beginning of the experiment. Nocturnal activity (night and dawn) of both species was confirmed.

## ﻿Introduction

Hornung (2011) and Tuf and Ďurajková (2022) comprehensively reviewed isopod adaptations contributing to colonisation of land. As water balance has great importance to woodlice, their physiological, morphological, and behavioural adaptive strategies have been extensively studied. Behavioural reactions are thought to contribute to the range of adaptations, both equally to morphological and physiological. Terrestrial isopods have developed various behavioural strategies to maintain water balance, such as pattern of activity, sheltering, and aggregation.

The avoidance of unfavourable environmental factors during the daytime via nocturnal activity was described by Cloudsley-Thompson (1952, 1956). It was suggested that conditions are more suitable at night due to lower temperatures, and, thereby, higher relative air humidity is favourable. The intensity of nocturnal activity is negatively correlated with the terrestriality of species, those better adapted to terrestrial conditions. *Armadillidiumvulgare* (Latreille, 1804) was found to be less active at night, with 60% of individuals nocturnally active, whereas *Philosciamuscorum* (Scopoli, 1763), which is thought to be less adapted, had 92% of individuals nocturnally active. During the daylight period, the isopods are expected to stay hidden in shelters to avoid desiccation.

Horizontal as well as vertical movements (Brereton 1957; Den Boer 1961; Tuf and Weissová 2022) enable finding suitable microhabitats. Behavioural responses to the environment, such as phototaxis, thigmokinesis, or hygrokinesis, are thought to stimulate movement. Photonegative orientation is associated with searching for dark habitats (shelters), which provide higher humidity and lower temperatures in comparison to the surrounding environment (Cloudsley-Thompson 1977).

Thigmokinetic reactions were linked with sheltering and aggregative behaviour by Allee (1926) and Broly et al. (2012). Thigmokinesis was characterised by Friedlander (1964) as a cessation of movement in response to contact stimuli. The fact that thigmokinetic reactions vary by individual was pointed out by Friedlander (1964) and Warburg (1968). This behaviour is supposed to strengthen or weaken spatial movement in response to environmental conditions.

### ﻿Sheltering

Hiding in shelters during the day to avoid predators or unfavourable environmental conditions such as low humidity has been extensively studied (Hornung and Warburg 1996; Dias et al. 2012), and a trade-off between time spent by foraging and sheltering was described by Dias et al. (2012). Woodlice species spend more time sheltering at a lower relative humidity (Dias et al. 2012; Dixie et al. 2015; Leclercq-Dransart et al. 2019; Delhoumi et al. 2023). There were also differences found in their main period of the year when they shelter. *Armadillidiumvulgare* preferred spring (March and April), whereas in *P.scaber* sheltering peaked during a pre-breeding period (May and June). Dangerfield and Hassall (1994) observed that males were more active and used artificial shelters less frequently than females, which possibly maximized foraging time and increased mating chance in May and June. Females were more often observed in shelters during the breeding season (June and July). For females, the shelters likely offered protection against desiccation and predation, and the higher average temperatures enabled shorter brood development time.

Kuenen and Nooteboom (1963) studied how three species, *Oniscusasellus* Linnaeus, 1758, *P.scaber*, and *A.vulgare*, react to external stimuli when searching for shelters at the end of their nocturnal activity. These authors suggested that olfactory signals influenced the individuals in addition to thigmotaxis and negative phototaxis, and they hypothesised that olfactory aggregation signals were supported by the presence of pheromones in faecal pellets, as known in several woodlouse species, including *P.scaber* and *A.vulgare* (Takeda 1980; Ebisuno et al. 1982).

### ﻿Aggregations

Aggregation, which usually occurs in shelters, enables animals to significantly reduce water loss. Increased aggregation was observed during dry conditions, and aggregated animals were found to evaporate 50% less water (Warburg 1968). Friedlander (1965) suggested that the aggregation stimuli could be influenced by contact (thigmokinesis) combined with special movements or a chemical signal. This social aspect of aggregation, probably related to pheromones, was studied in detail by Broly et al. (2012) who encountered a maximum aggregate of 70 woodlice. Despite the number and density of individuals of *P.scaber* in this experiment, the location of aggregates was determined by individual preferences. The dynamics of aggregation and collective choice were found to be controlled by social interaction between conspecifics.

Direct connections between water balance, temperature, light, and other factors have been examined through the lens of various behaviours, such as circadian activity (Cloudsley-Thompson 1956), sheltering behaviour (Den Boer 1961), aggregation (Allee 1926; Friedlander 1964; Broly et al. 2012), and movement activity (including phototaxes, thigmokinesis, hygrokonesis; Warburg 1993). On the other hand, Cloudsley-Thompson (1977) recommended a holistic approach, because various factors might be unknown, interacting in unexpected ways, and individuals could respond to them in various ways (Cloudsley-Thompson and Constantinou 1987). Since spatial movements are considered a significant behavioural adaptation to survive in terrestrial environment, a field study to explore locomotion and sheltering activity of terrestrial isopods was carried out.

We aim to evaluate (1) vagility of isopods by measuring the distance of woodlice from the releasing point, (2) sheltering behaviour of aggregations under artificial shelters, and (3) activity patterns, or numbers of isopods outside the shelters. The behaviour of two model species was compared.

## ﻿Materials and methods

### ﻿Model species, experimental site and design

Field experiments were carried out in 2011. The main part of the experiment was the monitoring of sheltering and movement of 1,000 marked individuals in the field, where a grid of artificial shelters was arranged. A pilot study had been conducted at the beginning of June. The main experiment was then redesigned based on the findings of the pilot study and run twice, once in June 2011 (Experiment 1) and once in September 2011 (Experiment 2). As a model species, *Armadillidiumversicolor* Stein, 1859 (Variegated Pill Isopod) and *Porcellioscaber* Latreille, 1804 (Common Rough Woodlice) were selected. Several comparative studies have examined abilities of woodlice species to survive in terrestrial conditions (Edney 1951; Schmidt and Wägele 2001). According to these studies, *A.vulgare*, which is considered comparable to *A.versicolor* (Csonka et al. 2013), was evaluated to be better adapted to the dry environment.

**Figure 1.**
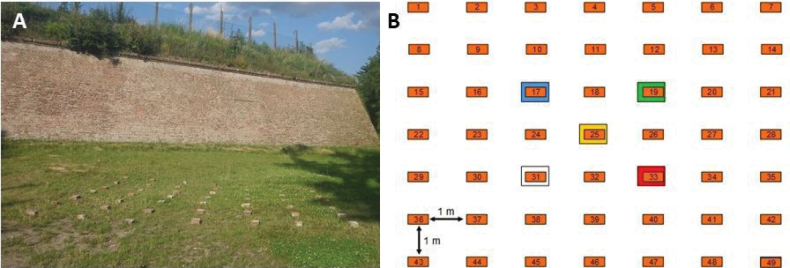
**A** grid of artificial brick shelters at experiment site nearby Korunní pevnůstka fortress (photograph RF) and **B** experimental design scheme with numbered orange bricks and releasing points of 200 coloured woodlice marked by larger boxes of relevant colours.

**Figure 2.**
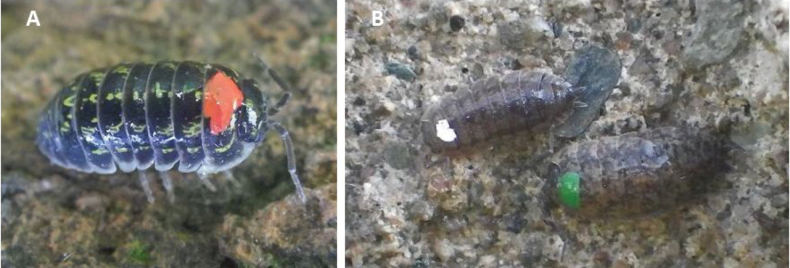
Marked individuals of **A***Armadillidiumversicolor* and **B***Porcellioscaber* (photographs RF).

Both species were collected in Olomouc City (Czech Republic) and kept in captivity for a few days before the experiment in large plastic boxes with soil and leaves, at a natural temperature, and natural light regime. Acetone-based mother-bee marking polish was used to identify five different colour groups (Fig. 1). These colour markings were used for quick counting of the animals during night inspections to reduce disturbance. White and green colours were used for *P.scaber*, and red, yellow, and blue colours were used for *A.versicolor*. There were 200 individuals marked for each colour group. Marking was done according to the method described by Drahokoupilová and Tuf (2012), who recommended making small, but clearly visible dots on dorsal sheet of the first segment of the pereion (Fig. 2). These markings had no negative effect on survival or feeding behaviour (Kenne et al. 2019) and is recommended for short-time field studies of dispersal of terrestrial isopods. Manipulation with individuals was quick and gentle to minimize stress, and specimens were marked 48 h prior to release.

The field study took place in a 36 m^2^ flat, square area in front of the Korunní Pevnůstka fortress in Olomouc, near a large park (49.5919°N, 17.2585°E). The study area consisted of a small grassy area with a regularly mowed lawn in front of the brick walls of the fortress. The site was selected as an optimal place for various terrestrial isopods, including *A.versicolor* and *P.scaber*, following Riedel et al. (2009), who described such a site as a natural habitat for various species of terrestrial isopods. The presence of bricks was also a consideration.

A grid of 49 shelters (7 × 7), each 1 m apart, was established to observe sheltering behaviour of 1,000 labelled animals. Bricks (29 × 15 × 6.5 cm) were used as shelters. Beneath each brick, a small space was dug out (15 × 10 × 1 cm) to avoid killing animals when manipulating the brick. Shelters had been put in place 10 days before the release of animals. Five shelters were used as release points when the experiment started. Each colour group (200 specimens) was released under one shelter (Fig. 1).

The study site was visited regularly three times each night: at dusk, in the middle of the night, and at dawn. In Experiment 1, isopods were monitored for three nights (June 24–27, 2011, checks at 1:00, 5:00, 21:00) and in Experiment 2 for seven nights (September 22–29, 2011, checks at 0:00, 7:00, 19:00). Each shelter was briefly opened (the brick was lifted), and the number and identity (colour group) of individuals present were recorded by a camera using weak, white illumination. Cole’s comments (Cole 1946) on the counting procedure, that is, count animals on the ground surface and on the underside of the brick, do not count animals hidden in soil crevices, were following.

### ﻿Data processing and analysis

Data sets from each monitoring check were processed according to the colour groups and species. The individual records were then matched with the distances achieved from the release point, distances achieved for each colour group, and then the distance for each species was calculated.

The Wilcoxon nonparametric paired test was used to compare vagility of two species and, therefore, two equal groups were needed. This meant comparing only 400 individuals of *A.versicolor* with the 400 individuals of *P.scaber*. The *Armadillidium* colour group selection was carried out according to the sum of the average distances (in meters) of two colour groups, the middle sum was chosen as a representative. Blue and red colour groups were selected by this method.

Firstly, it was necessary to count subtractions of mean achieved distances for each night (“dusk distance mean” minus “dawn distance mean”). The limit of 10 individuals was set to decrease random effects. These figures were counted for each species. Secondly, obtained figures were used for the Wilcox nonparametric paired test. Experiments 1 and 2 were tested separately. Prism 6 software by GraphPad Software Inc was used to compute the test.

The effect of activity pattern of woodlice was tested by evaluating the abundances in the three periods during the night (dusk, night, and dawn). Because time was expected to significantly affect the abundances, the generalized linear mixed model (GLMM) was used to justify effect of two fixed variables, such as time (quantitative: 1–3 in Experiment 1, 1–7 in Experiment 2) and day period (categorical: dusk, night, or dawn). It was set Poisson error distribution and logarithmic link function to model the effect of both predictors. While creating the model, we considered predictor time for replication because of the repeated measurements. SAS v. 9.2 (SAS Institute Inc. 2007) was used for the statistical analysis.

To enable comparison, blue and red groups for *A.versicolor* were used to create graphs based on similar process, as described above for vagility comparison.

## ﻿Results

### ﻿Movement

In both experiments, shelters at distances of 1–5 m from the release point were inhabited by specimens of both species after 20 h. The maximum recorded distance was 5.56 m travelled by *P.scaber* in 5 h and the same distance was travelled by *A.versicolor* in 12 h in Experiment 2 (Table 1).

**Table 1.** Maximum observed distance (m) measured from releasing point achieved by isopods during start of experiment.

Experiment 1			Experiment 2		
Time	*P.scaber*	*A.versicolor*	Time	*P.scaber*	*A.versicolor*
4 h	4.12 m	4.47 m	5 h	5.56 m	3.16 m
20 h	4.47 m	4.47 m	12 h	5.56 m	5.56 m
24 h	5.56 m	4.47 m			
28 h	5.56 m	5.56 m			

### ﻿Vagility

**Figure 3.**
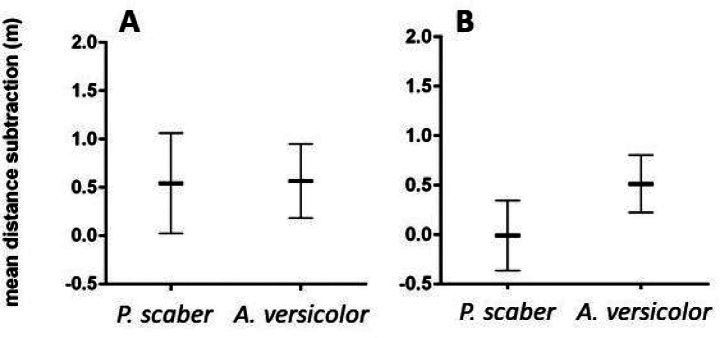
Species vagility comparison of results represented by mean distance subtractions (mean ± SE) during **A** Experiment 1 and **B** Experiment 2.

There was no significant difference in vagility between the two species (Fig. 3). In Experiment 1, vagility of both species was similar (mean_P.scaber_ = 0.54 m, mean_A.versicolor_ = 0.57 m; *p*_Exp1_ > 0.9999). Non-significantly higher vagility of *A.versicolor* was observed in experiment 2 (mean_P.scaber_ = 0.007 m, mean_A.versicolor_ = 0 .5140 m; *p*_Exp2_ = 0.0938).

### ﻿Sheltering pattern

**Figure 4.**
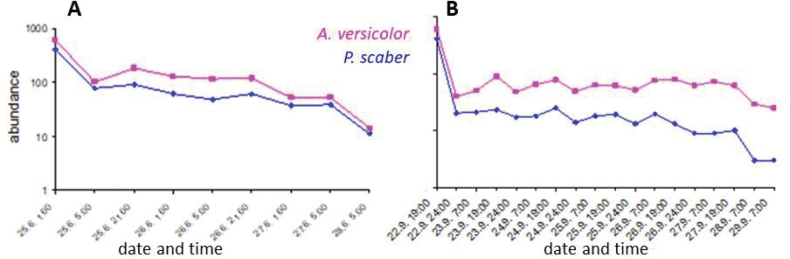
Number of re-detected individuals according to monitoring time controls (note the logarithmic scale of the Y-axis) in **A** Experiment 1 and **B** Experiment 2.

**Figure 5.**
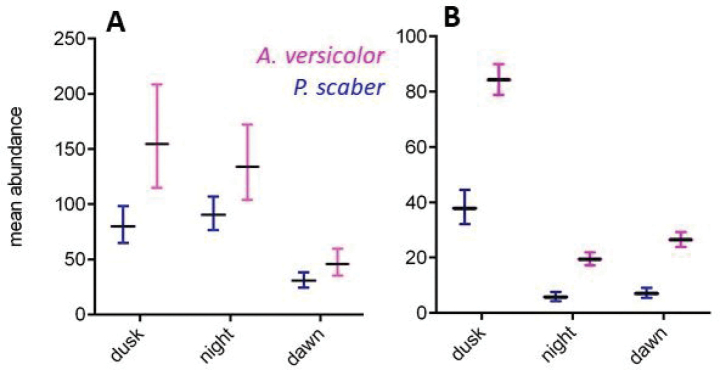
Influence of day period (dusk, night, and dawn) on number of woodlice (mean ± SE) in shelters during **A** Experiment 1 and **B** Experiment 2.

**Figure 6.**
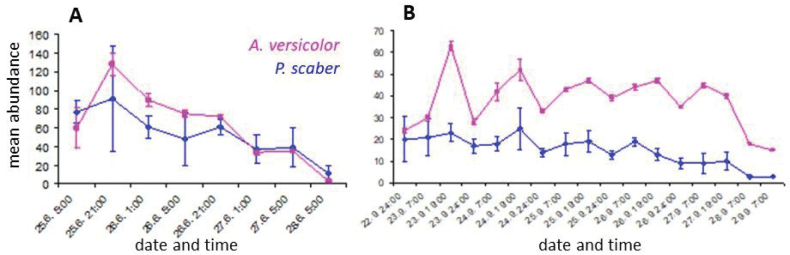
Comparison of species abundance (mean ± SE) based on number of individuals aggregated under one brick in **A** Experiment 1 and **B** Experiment 2.

Abundance of sheltering individuals differed in each experiment, as well between the species (Figs 4, 5). Individuals were more abundant in Experiment 1 (289 pillbugs and 179 woodlice recorded in the 1^st^ and 2^nd^ evening), compared to 111 and 102 individuals in Experiment 2. Individuals of *A.versicolor* were more frequently detected than *P.scaber* (Fig. 6).

Time (each night was considered to be a category of time) was confirmed to negatively affect the abundance of individuals observed under the shelters (Fig. 4) as the individuals of both species dispersed out of the experimental area (Experiment 1: F_P.scaber_ = 333.93, p_P.scaber_ < 0.001; F_A.versicolor_ = 500.64, p_A.versicolor_ < 0.001; Experiment 2: F_P.scaber_ = 477.14, p_P.scaber_ < 0.001; F_A.versicolor_ = 411.49, p_A.versicolor_ < 0.001). In Experiment 1, 1,000 individuals were released and 198 individuals were observed 24 h later; in Experiment 2 only 111 remained after the same time. The small number of marked individuals found (25 and 28 in Experiments 1 and 2, respectively) limited the duration of the experiment.

Significant differences in abundances between the day periods (dusk, night, and dawn) were found (Experiment 1: F_P.scaber_ = 77.27, p_P.scaber_ < 0.001; F_A.versicolor_ = 128.54, p_A.versicolor_ < 0.001; Experiment 2: F_P.scaber_ = 195.35, p_P.scaber_ < 0.001; F_A.versicolor_ = 240.80, p_P.versicolor_ < 0.001). In comparing abundances according to day period, the results between experiments differed (Fig. 5). The results also varied between species in Experiment 1. Dusk was found to be the main sheltering period for *A.versicolor* in Experiment 1 and for both species in Experiment 2. Night was least popular for sheltering for both species in Experiment 2. This contrasted with the behaviour of *P.scaber* in Experiment 1, where night was most popular for sheltering. Dawn was the least frequent sheltering period in Experiment 1 for both species.

Shelters were not inhabited equally, and their use varied in both time and in aggregation size. Some shelters were never inhabited (Experiment 1: bricks 11, 21, 36; Experiment 2: bricks 6, 45, 47), whereas individuals of all five colour groups were observed at the same time under some shelters (Experiment 1: bricks 6, 16, 20, 27, 36; Experiment 2: bricks 15, 24).

**Figure 7.**
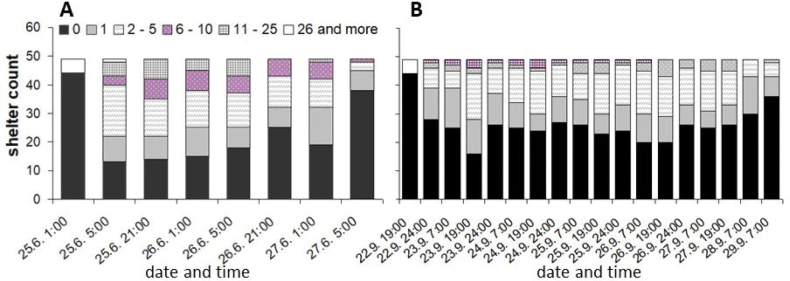
Frequency of aggregation of different size categories (according to sum of individuals of both woodlouse species aggregated) observed under each shelter in **A** Experiment 1 and **B** Experiment 2.

The sum of individuals under each shelter (aggregation size) varied among shelters and according to time. The aggregation size was categorised according to the sum of individuals (categories: 0, 1, 2–5, 6–10, 11–25, 26 and more; Fig. 7). The frequency of observing empty shelters increased as time progressed. Aggregations of 2–5 individuals were more frequent than one individual, mainly in Experiment 1. Aggregations of 6–10 individuals were observed in almost every check, except the last one. The very large aggregations of 11–25 individuals were found under some shelters (Experiment 1: bricks 12, 19, 20, 25, 31; Experiment 2: bricks 10, 19, 24).

## ﻿Discussion

We studied the movement and sheltering behaviour of *Porcellioscaber* and *Armadillidiumversicolor* in a field experiment. Changes in distribution of marked individuals in artificial shelters were monitored during three nights in June and eight nights in September.

### ﻿Dispersal activity

Brereton (1957) noted that *P.scaber* was not nomadic and the same individuals were found on the same tree for a long time. On the other hand, he also recorded some immigrants in his study. Paris and Pitelka (1962) stated that the population of *A.vulgare* was very mobile; they released 243 marked animals but only eight animals (3.3%) were recaptured 24 hours later within a circular area (12.5 m^2^) with radius of 2 m. In our study, the recapture rates were higher; of the 1,000 individuals released in an area of 36 m^2^, 19.8% were observed 24 h later in Experiment 1 and 11.1% in Experiment 2 . However, our study is not entirely comparable because the isopods were sampled only under artificial shelters. In another study, Paris (1965) labelled 1,000 *A.vulgare* by using the P^32^ isotope and released them in a circular grid of artificial shelters. He detected them at a distance of 1 m within 4 min and 3 m within 50 min. The maximum dispersal rate was 13 m in 12 h and maximum distance of 25 m was observed on day 20; greater dispersal rates were observed during a dry season. Our observed distances of 4.4 m/4 h (*A.versicolor*) and 5.6 m/5 h (*P.scaber*) were consistent with Paris’s (1965) field experiment.

Other factors influencing natural behaviour, such as colour marking, should be also considered, as they might negatively affect woodlice activity (Drahokoupilová and Tuf 2012; Kenne et al. 2019). Translocation and handling might also negatively influence movement behaviour. Heidinger et al. (2009) found that animals dispersed over longer distances in the first day after release and tended to move further after release in general. Therefore, it is highly probable that woodlice do not usually move as far as observed in our study unless disturbed.

### ﻿Vagility

We found no significant differences in vagility between the two species, although we observed slightly a higher vagility rate for *A.versicolor* in Experiment 2. These results do not support the hypothesis by Dailey et al. (2009), who suggested that *A.vulgare*, as a conglobating isopod (Schmalfuss 1984), relies on passive defence, whereas *P.laevis* uses active escape strategies for avoiding harsh conditions and predators. The hypothesis of higher vagility in *P.scaber* might be consistent with its lower abundance in our experiments, as compared to *A.versicolor*. In our experiments a greater number of *P.scaber* individuals had run away from the experiment area and, thus, they were not recorded. It is possible that a decline in the abundance of *P.scaber*, probably caused by migration away from the site, is indirect evidence of the higher vagility of this species, which was underestimated in the design of the study area’s of size.

### ﻿Sheltering behaviour

Abundances of shelter inhabitants were uneven, similar to the other studies (Davis and Sutton 1977; Hornung and Warburg 1996). Den Boer (1961) suggested that good hiding sites were occupied by many individuals every night because of better conditions in the shelters and due to active searching behaviour of woodlice for exactly such conditions. Hornung and Warburg (1996) proposed that moisture conditions in the shelters can be important in the uneven distribution. This explanation is not supported by recent studies, where the presence of conspecifics is much more important for sheltering of *P.scaber* than the quality of shelter alone (Devigne et al. 2011; Broly et al. 2012; Zidar and Fišer 2022). This explanation is consistent with olfactory effects of an aggregation pheromone (Kuenen and Nooteboom 1963; Takeda 1980; Ebisuno et al. 1982). Moreover, the aggregation pheromone attracted individuals of woodlice of the same species as well as other terrestrial isopods. This likely means that the empty shelters did not offer attractive conditions and were observed more often than the shelters with at least one individual. The individuals tend to join others in already inhabited hiding sites (Hassall and Tuck 2007) in accordance with the hypothesis that the risk of poor conditions at the site where many other conspecifics have already aggregated is lower. Therefore, the shelter that offered suitable conditions and had been found was more likely to be the one used throughout the whole monitoring period.

Broly et al. (2012) discovered the social aspect of aggregation behaviour of *P.scaber*, noting 60–70 individuals per aggregation as the maximum. The number of individuals found in shelters in our field study was usually much lower, but we counted isopods only during the night when locomotory activity outside the shelters usually occurs.

### ﻿Temporal pattern of sheltering behaviour

Our woodlice were outside shelters mainly during the night and dawn in Experiment 2, which is in accordance with other studies on *P.scaber* (Den Boer 1961), *A.vulgare* (Cloudsley-Thompson 1952, 1956; Paris 1963; Refinetti 2009), and *A.versicolor* (Tuf and Weissová 2022). Temperature and humidity were not recorded during our study, but it is expected that, by dawn, the humidity of the air increased as the temperature dropped; these are conditions favourable for woodlice foraging. The results of Experiment 1 do not fit the described pattern of night activity, as the experiment was short and, therefore, more affected by the high woodlice activity after their release (Heidinger et al. 2009).

The results of this study suggest that terrestrial isopods can use artificial shelters in natural conditions at least for short research periods. Over the course of several nights, the number of hiding woodlice was observed, and this pattern may be due to increased locomotor activity outside of the shelters, as well as to the animals moving away from the experiment area or using natural shelters in the soil. Our study confirms the temporal activity patterns of woodlice, the abundance of individuals in the shelters, and the simultaneous use of shelters by the two species. For more detailed studies of shelter fidelity and experiments with individual marking of animals will be necessary.

## ﻿Additional information

### Author ORCIDs

Ivan Hadrián Tuf https://orcid.org/0000-0003-0250-0482

### Data availability

All of the data that support the findings of this study are available in the main text.
